# Some Observations on the Connexion of the New and Full Moon with the Invasion and Relapse of Fevers

**Published:** 1787

**Authors:** Robert Jackson

**Affiliations:** Physician at Stockton, in the County of Durham


II. *Some Obſervations on the Connexion of the new and full Moon with the Invaſion and Relapſe of Fevers. By* Robert Jackſon, *M. D. Phyſician at Stockton, in the County of Durham. Communicated in a Letter to Sir* Joſeph Banks, *Bart. P. R. S., and by him to Dr.* Simmons.

*To Sir* JOSEPH BANKS, *Bart. P. R. S.*

SIR,

THERE is a ſubject which at preſent ſeems, in ſome degree, to engage the attention of the Public — I mean the influence, or, to ſpeak more circumſpectly, the connexion of the new and full moon with the invaſion and relapſe of fevers. It is now about a dozen years ſince I made ſome obſervations on this matter in the Weſt Indies, and probably I ſhould have made them known to the world before now, had I been able to have carried them on in the extenſive manner I originally intended; but the neceſſity of attending to ſome concerns of life has obliged me to leave the deſign, if not totally, in a great meaſure unfiniſhed. In the Weſt Indies, indeed, in the iſland of Jamaica, I think I may ſay my obſervations were made with care. The ſame care was con-

 tinued tinued in the fouthern provinces of America; but my papers, with the original remarks, having been loft in one of our unfortunate rencounters with the enemy, I can only charge my memory at this diftance of time with what was the general refult of them. In France, Germany, and Italy, I had made a few curfory remarks; not numerous, or pointed enough, however, to be depended on. In England I can fay nothing, my opportunities of obferving here having hitherto been very confined.

Within thefe two years the world has been favoured with a treatife on this fubject by Dr. Balfour, a gentleman who has refided and practifed feveral years in India: but his account differing materially from what was obferved by me in the Weft Indies and in America, I take the liberty, Sir, of prefenting to you the refult of my inquiries on this fubject, without any other apology than what the importance of the fubject carries along with it. It is a fubject which I am perfuaded will be curious to you as a philofopher; and the knowledge of it may be of ufe to the phyfician. This, then, being the cafe, I fhall beg leave to mention to you in a few words, and a very few words will fuffice,

ſuffice, how the idea aroſe, and the manner in which it was proſecuted.

I went out to the Weſt Indies in the beginning of the year 1774. I was apprized of what Dr. Lind had mentioned, as an effect of the new and full moon, and of eclipſes, on the relapſe of fevers in India, and might be ſuppoſed not unprepared to expect ſomething of a ſimilar kind in Jamaica. Accordingly, before the end of the year, I had reaſon to believe it was a thing even in that country not without foundation. In the courſe of the year following I went farther — I obſerved that frequently three or four of a company of ſoldiers, who were quartered at the place where I reſided, and of whom I had the care, fell ill on the ſame day; and that this did not happen, perhaps, above once in a fortnight. The thing ſeemed curious; and as it happened repeatedly at the time the moon was near full, a hint was ſuggeſted, that not only the relapſe, but that the firſt attack of fever was probably connected with the changes of that planet. To aſcertain the truth of it, however, in the beginning of the year 1776 I provided myſelf with an almanack, and on the blank leaves of it marked the preciſe date of attack of ſuch

 fevers fevers as came under my care. At the end of the year, of thirty cafes of proper remitting fever, I found that twenty-eight had happened on one or other of the feven days preceding a new or full moon. The year following, of twenty-eight, there were only twenty-two: it was remarked, however, that of thofe fix which happened in the period which follows new and full moon, three had happened on the day of the new moon, and only a few hours after the change had taken place. Befides thofe cafes of proper fever, I had taken notice of a number of day fevers and flight feverifh diforders, the attack of which feemed likewife to have been influenced by the fame caufe, though in a fmaller degree.

This precifely is the cafe as it ftood in the almanacks, accompanied, however, with a few remarks and explanations; the principal of which are the following: — That though the feven days preceding new and full moon, or the fecond and laft quarters, are what might be reckoned the fickly period, yet it was in the four days immediately preceding that the attack of fevers was chiefly remarkable; that the invafion of fevers was always nearer new or full moon in the dry and healthy feafon, than in the rainy months, when diſeaſes were common, particularly when they were epidemic; that this was likewiſe more obſervable in the mild, remitting fevers, than in thoſe of a violent and malignant kind; and in the ſoldiers of the garriſon, who were expoſed to few occaſions of diſeaſe, than in the inhabitants of the town and country, whoſe various employments ſubjected them oftener to fatigues, or carried them oftener to unhealthy ſituations.

The above obſervations, which were made at Savannah la Mar, in Jamaica, ſeeming to carry with them a very decided proof of the influence, or connexion of the moon with the invaſion and relapſe of fevers, I thought it might be of conſequence to proſecute the idea in the different countries I might happen to viſit. In the year 1778, I joined the army in America, and went to ſerve in a regiment that was quartered on York iſland. In the months of June and July, the regiment being encamped in a dry and healthy ſituation, fevers were rare; and ſuch as happened, were in the period approaching to a new or full moon. In Auguſt, it was removed to Kingſbridge, and encamped in the neighbourhood of ſome low and marſhy ground, where it continued the whole of the autumn autumn. An intermitting fever ſoon began to appear, and ſoon became highly epidemic: its time of invaſion, with reſpect to the moon, was greatly more irregular than it had been in the former months; at the end of the year, however, of one hundred caſes, about eighty were found to be in the period above mentioned. What ſeemed remarkable, relapſes were rather in a ſmaller proportion. In the years 1779, 1780, and 1781, the regiment ſerved in the ſouthern provinces, and may be ſaid, indeed, to have been almoſt conſtantly in the field: it was often encamped in unhealthy ſituations, and often had the intermitting fever epidemic in a high degree. When that was the caſe, as was obſerved before, the irregularity of invaſion was greater; yet even then, the approach to new and full moon ſeldom failed to double the number of the ſick: but my memorandums having been loſt, I cannot exactly aſcertain the proportion in the three laſt campaigns.

Having related the above obſervations, which I flatter myſelf were made in a manner little liable to deception, and in the noting of which I was not conſciouſly biaſſed by theory, it may not be amiſs to take a view of the account given by Dr. Balfour. The three days immediately prepreceding, and the three days immediately following, new and full moon, are what are mentioned by him, as the period remarkable for the invaſion and relapſe of fevers: but it is pity, he does not tell us on what facts he founded this opinion. If it is drawn from a looſe, from a groſs eſtimate of what he thought he ſaw, it cannot be depended on. Unleſs a man is circumſtantial in his facts, and very circumſtanſtantial too, a preconceived opinion leads him aſtray; for it is ſeldom, very ſeldom, that theory does not run before obſervation. What he obſerves, indeed, it muſt be allowed, is more agreeable to what we ſuppoſe to be the effect of the moon on the tides: but the caſe here ſeems widely different; and, probably, were we to reaſon on a ſubject that ought not, indeed, to be made the ſubject of reaſoning, we might find ſome explanation of it. It being agreed on both ſides, that the new and full moon are to be conſidered as a powerful exciting cauſe of fever, it is more reaſonable to ſuppoſe, that that cauſe will produce its effect while it is acquiring vigour, rather than while it is loſing it; that is, in the days that precede the new and full moon than in thoſe that follow: but this is only a ſuppoſition of what may be the cauſe; the fact is certain, as far as a fact in physic can well be. And were any more proofs wanted, to the above we might add the different periods at which fevers have a tendency to relapse. In the West Indies, and in America, particularly in the West Indies, where the crisis for the most part was the work of nature, fevers, when they did return, almost always returned on a seventh, a fourteenth, a twenty-first, or a twenty-eighth day from the termination of the fever; more of them, indeed, on the fourteenth than on all the others put together. This observation was not unknown to many practitioners in Jamaica; it was common with them to say, when a person had had a bad fever, that he would either have a return of it in a fortnight, or some other disease; and the prediction in the course of three or four years was seen seldom to fail.

The above facts having put it beyond a doubt, that the new and full moon, or the approach to new and full moon, is a powerful exciting cause of fever, it would be a matter of much consequence to determine the degree of it in the different parts of the world. Surgeons of regiments, and those who have the care of convents and hospitals, have the best opportunity nity of coming at the truth. Obfervations made on the people at large, who live in a thoufand different ways, will always be uncertain; and the accounts that phyficians receive of difeafes that they do not fee till a late period, are not at all times to be depended on. The importance of the fubject is fuch, that it well deferves to be inquired into. It particularly concerns the army; and it is no rafh affertion to fay, that a knowledge of this principle, and a knowledge of the proper ufe of bark, will go farther in preferving the health of an army, an army on fervice, than all the other helps of medicine put together. Of this I had a ftrong proof during the campaigns of 1780 and 1781, in the corps I had the honour to ferve in.

I am, with great refpect,

SIR,

Your moft obedient

Humble fervant,

ROBERT JACKSON.

*Stockton,*

Dec. 3, 1786.

